# Speaking and cognitive distractions during EEG-based brain control of a virtual neuroprosthesis-arm

**DOI:** 10.1186/1743-0003-10-116

**Published:** 2013-12-21

**Authors:** Stephen T Foldes, Dawn M Taylor

**Affiliations:** 1 Department of Biomedical Engineering, Case Western Reserve University, Cleveland, Ohio 44106, USA; 2 Cleveland Functional Electrical Stimulation (FES) Center of Excellence, Louis Stokes VA Medical Center, Cleveland, Ohio 44106, USA; 3 Department of Neurosciences, The Cleveland Clinic, Cleveland, Ohio 44195, USA

**Keywords:** Brain-computer interface (BCI), Electroencephalography (EEG), Neuroprosthesis, Cognitive load, Functional electrical stimulation (FES), Brain-machine interface (BMI)

## Abstract

**Background:**

Brain-computer interface (BCI) systems have been developed to provide paralyzed individuals the ability to command the movements of an assistive device using only their brain activity. BCI systems are typically tested in a controlled laboratory environment were the user is focused solely on the brain-control task. However, for practical use in everyday life people must be able to use their brain-controlled device while mentally engaged with the cognitive responsibilities of daily activities and while compensating for any inherent dynamics of the device itself. BCIs that use electroencephalography (EEG) for movement control are often assumed to require significant mental effort, thus preventing users from thinking about anything else while using their BCI. This study tested the impact of cognitive load as well as speaking on the ability to use an EEG-based BCI.

**Findings:**

Six participants controlled the two-dimensional (2D) movements of a simulated neuroprosthesis-arm under three different levels of cognitive distraction. The two higher cognitive load conditions also required simultaneously speaking during BCI use. On average, movement performance declined during higher levels of cognitive distraction, but only by a limited amount. Movement completion time increased by 7.2%, the percentage of targets successfully acquired declined by 11%, and path efficiency declined by 8.6%. Only the decline in percentage of targets acquired and path efficiency were statistically significant (p < 0.05).

**Conclusion:**

People who have relatively good movement control of an EEG-based BCI may be able to speak and perform other cognitively engaging activities with only a minor drop in BCI-control performance.

## Findings

### Introduction

Users of BCI-controlled devices, such as an upper-limb neuroprosthesis [1-4], must be able to use their device while talking and performing other cognitive tasks. Talking could potentially degrade EEG-controlled BCIs due to power spectral changes associated with verbal and cognitive engagement and the large electrical signals from muscles under the scalp. However, EEG-based BCI systems are usually evaluated with the subjects sitting quietly and focusing exclusively on the BCI task. This study tested the effects of speaking and cognitive load on the ability to command an upper-limb neuroprosthesis using EEG. All study activities were approved by the Institutional Review Board of the Louis Stokes Cleveland Veteran’s Affairs Medical Center.

### Methods

**Figure 1:**
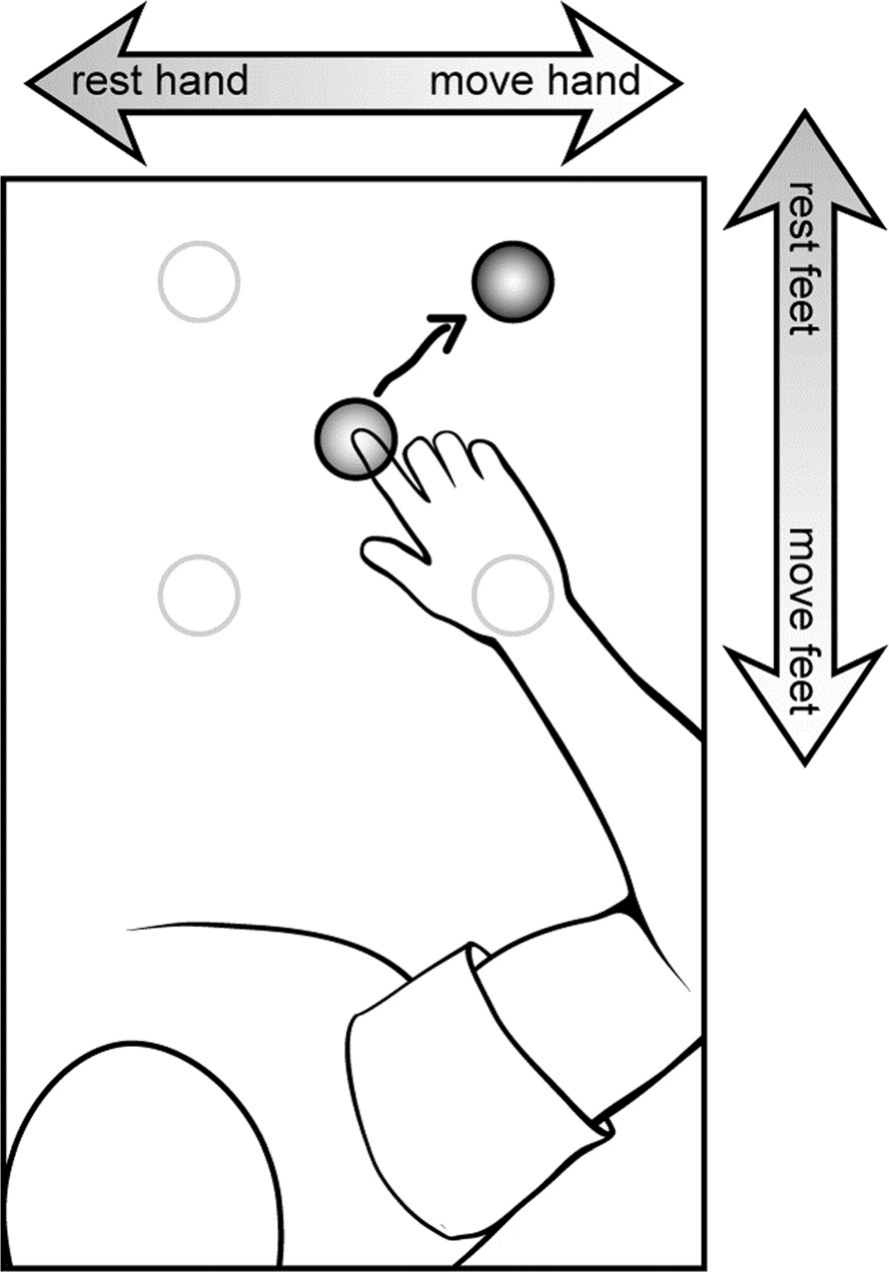
**Actions used to control the 2D velocity of the virtual neuroprosthesis on the screen.** Participants commanded the continuous 2D velocity of the fingertip of the virtual arm as it moved along a table top in a center-out-and-hold task. EEG changes associated with varying degrees of physically moving/resting the feet were used to command the virtual fingertip proximally-distally, while the degree of physically moving/resting the right hand was used to command the virtual fingertip right-left.

Six able-bodied individuals with little or no prior BCI experience used either 16 or 32 channels of EEG to control a virtual neuroprosthesis arm with realistic dynamics in a 2D reach-and-hold task along a tabletop. Changes in mu and beta power associated with hand and feet movements were used to generate proportional 2D velocity commands in real time. Decoding functions (least-squares regression) and spatial filters (2D common spatial pattern) were generated from 5.3 minutes of actual, open-loop movements (methods detailed in [5]). Figure 1 illustrates how EEG modulations associated with different degrees of physically moving/resting the right hand or both feet were translated into a proportional 2D velocity command. This study included only participants with above-chance level EEG modulation associated with both hand and foot movements. Velocity commands were used to control the continuous motion of the fingertip of the virtual neuroprosthesis viewed on a computer screen as if looking down on the arm from above. Realistic dynamics of a paralyzed arm activated via electrical stimulation were added to the virtual neuroprosthesis using previously reported methods [6,7].

Participants performed a ‘center-out-and-hold’ task where they had 12 seconds to move a cursor located at the fingertip of the virtual arm from the center of the workspace to one of four radial targets. Participants had to keep the fingertip/cursor touching the target for one second for the trial to count as a success.

To assess the effects of performing cognitive tasks and speaking on movement control, participants were tested under three levels of cognitive load (CL), two of which involved speaking. Random letters were played out loud every two seconds as participants performed the center-out-and-hold task. Participants were instructed to either: 1) only focus on controlling the virtual neuroprosthesis arm (‘No CL’), 2) repeat each letter immediately after hearing it (‘Moderate CL’), or 3) remember and repeat the previous letter immediately after hearing the current letter in a ‘one-back’ fashion (‘High CL’). The second and third conditions both required speech encoding and production but engaged working memory to different degrees [8]. Subjects five and six were asked to speak each cued letter five times instead of once to generate more speech-related activity while maintaining the same cognitive load. Subjects five and six also only used 16 instead of 32 electrodes distributed over the same area due to connector issues.

Letters were played during the ‘No CL’ condition to ensure the auditory environment was the same across all tests. No speaking or cognitive tasks were performed during the initial open-loop data collection that was used to build the decoder. After one block of practice, each participant generated at least 40 center-out-and-hold movements under each of the three conditions. The order of test conditions was varied across subjects and each subject repeated their sequence of conditions twice to minimize learning and fatigue confounds.

Performance was assessed with three metrics: a) the percentage of trials that were successful, b) the average time required to successfully reach a target, and c) the average path efficiency (i.e. shortest path to the target divided by the length of the actual path taken).

### Results

**Figure 2:**
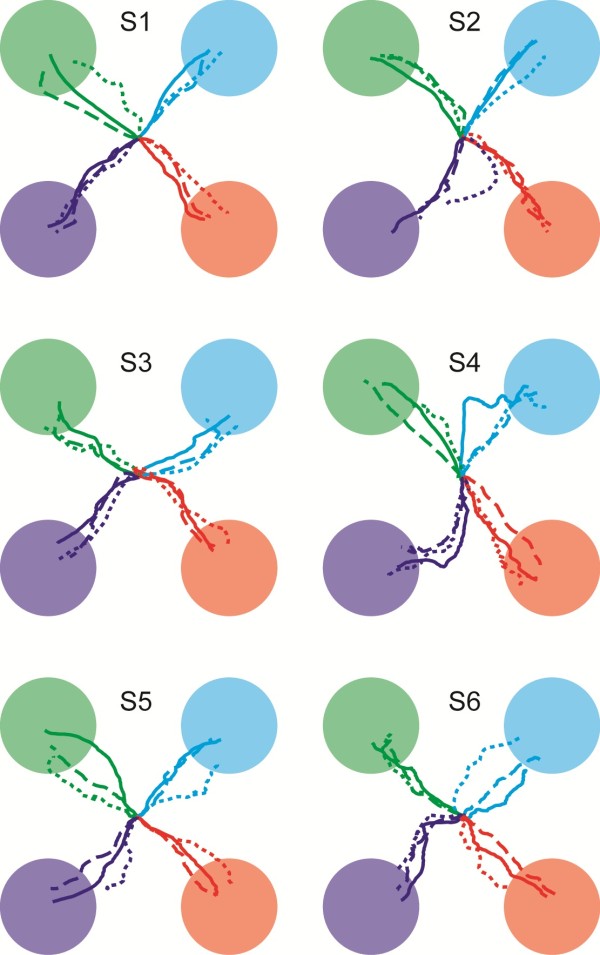
**Average of all brain-control movement trajectories for each subject under different levels of cognitive load.** Solid lines = No CL, dashed lines = moderate CL, and dotted lines = high CL. Colored circles represent the area in which the fingertip had to stay for one second to count as a hit (i.e. circles have a radius equal to the target radius plus the radius of the fingertip cursor).

Mean movement trajectories for each participant under each cognitive load condition are shown in Figure 2. On average, similar directional control was seen across all three cognitive load conditions.

**Figure 3:**
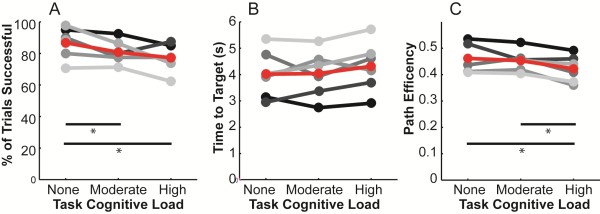
**Quantitative effects of cognitive load and talking on EEG-control of a virtual neuroprosthesis.** Lines are shaded by subject number as listed in Figure 2 (black = 1, lightest grey = 6). Red line indicates the mean across subjects. Asterisks indicate significance of p < 0.05. **A)** the percentage of trials that were successful. **B)** the average time to successfully reach the target. **C)** the average path efficiencies.

Performance metrics for the BCI task are shown in Figure 3. On average, all metrics showed a decline in control performance with increased cognitive load, although this decline was not significant in the more variable movement-time metric (paired t-test).

### Discussion

This study sought to quantify the impact of additional cognitive load on an EEG-BCI where participants used different combinations of hand and foot movements to command the 2D velocity of the fingertip of a virtual neuroprosthesis. Our results demonstrated that the added cognitive load imposed by the letter repetition tasks produced only a moderate drop in control performance on average. However, given the added challenges of using different types of BCIs, it is important to further characterize how cognitive tasks and speech production impact BCI performance.

We have shown previously that strong jaw muscle activity associated with teeth clenching can be detected on EEG electrodes across much of the scalp [9]. Therefore we anticipated that jaw muscle activity associated with talking might broadly increase the power in the recorded signals during the moderate-CL and high-CL tasks. Such an increase in power would have resulted in a command bias toward the left-distal direction because that is the direction associated with increased mu and beta power due to resting the hands and feet. However, the trajectories in Figure 2 did not show any systematic skewing toward the left-distal part of the workspace. Therefore, these results suggest the muscle activity required for simple speech production was not enough to cause a significant bias in directional control. However, additional studies are needed to determine if more animated speech, facial expressions, and additional cognitive burdens (e.g. mental calculations, emotions) may still disrupt BCI use.

The ability to use an EEG-BCI can vary widely between individuals [10]. This study only included participants that naturally had movement-related modulation because it is likely that relatively good modulation will be needed for an individual to adopt a BCI into their daily life. People with more difficulty using a sensorimotor-rhythm-based BCI may have more problems performing cognitive tasks during BCI use. It is expected that these users could improve their control with additional training [3]. However, further studies are needed that include individuals with paralysis and people who have improved their EEG modulation with training to fully assess the practicality of BCI technology for a broad range of users.

## Competing interests

The authors declare that they have no competing interests.

## Authors’ contributions

SF participated in the design of the study, collecting and analyzing data, and drafting the manuscript. DT participated in the conception and design of the study, collecting and analyzing data, and drafting the manuscript. Both authors read and approved the final manuscript.
